# Predicting “When” in Discourse Engages the Human Dorsal Auditory Stream: An fMRI Study Using Naturalistic Stories

**DOI:** 10.1523/JNEUROSCI.4100-15.2016

**Published:** 2016-11-30

**Authors:** Katerina Danae Kandylaki, Arne Nagels, Sarah Tune, Tilo Kircher, Richard Wiese, Matthias Schlesewsky, Ina Bornkessel-Schlesewsky

**Affiliations:** ^1^Department of Psychiatry and Psychotherapy, University of Marburg, 35039 Marburg, Germany,; ^2^Department of Germanic Linguistics, University of Marburg, 35032 Marburg, Germany,; ^3^Department of Bioengineering, Imperial College London, London SW72AZ, United Kingdom,; ^4^Institute of Psychology I, University of Lübeck, 23562 Lübeck, Germany, and; ^5^Cognitive Neuroscience Laboratory, School of Psychology, Social Work and Social Policy, University of South Australia, Magill Campus, Adelaide SA 5001, South Australia, Australia

**Keywords:** auditory, dorsal stream, fMRI, language, predictive coding, temporal receptive windows

## Abstract

The hierarchical organization of human cortical circuits integrates information across different timescales via temporal receptive windows, which increase in length from lower to higher levels of the cortical hierarchy (Hasson et al., 2015). A recent neurobiological model of higher-order language processing (Bornkessel-Schlesewsky et al., 2015) posits that temporal receptive windows in the dorsal auditory stream provide the basis for a hierarchically organized predictive coding architecture (Friston and Kiebel, 2009). In this stream, a nested set of internal models generates time-based (“when”) predictions for upcoming input at different linguistic levels (sounds, words, sentences, discourse). Here, we used naturalistic stories to test the hypothesis that multi-sentence, discourse-level predictions are processed in the dorsal auditory stream, yielding attenuated BOLD responses for highly predicted versus less strongly predicted language input. The results were as hypothesized: discourse-related cues, such as passive voice, which effect a higher predictability of remention for a character at a later point within a story, led to attenuated BOLD responses for auditory input of high versus low predictability within the dorsal auditory stream, specifically in the inferior parietal lobule, middle frontal gyrus, and dorsal parts of the inferior frontal gyrus, among other areas. Additionally, we found effects of content-related (“what”) predictions in ventral regions. These findings provide novel evidence that hierarchical predictive coding extends to discourse-level processing in natural language. Importantly, they ground language processing on a hierarchically organized predictive network, as a common underlying neurobiological basis shared with other brain functions.

**SIGNIFICANCE STATEMENT** Language is the most powerful communicative medium available to humans. Nevertheless, we lack an understanding of the neurobiological basis of language processing in natural contexts: it is not clear how the human brain processes linguistic input within the rich contextual environments of our everyday language experience. This fMRI study provides the first demonstration that, in natural stories, predictions concerning the probability of remention of a protagonist at a later point are processed in the dorsal auditory stream. Results are congruent with a hierarchical predictive coding architecture assuming temporal receptive windows of increasing length from auditory to higher-order cortices. Accordingly, language processing in rich contextual settings can be explained via domain-general, neurobiological mechanisms of information processing in the human brain.

## Introduction

Human communication is shaped fundamentally by our linguistic abilities. Nevertheless, and despite considerable progress in characterizing the functional neuroanatomy of language processing (for a recent review, see Price, 2012), we still know very little about how the human brain implements the extremely rich discourses, narratives, and texts that form part of our everyday language experience. Developing neurobiologically plausible models at this rich contextual level is particularly challenging.

**Figure 1.**
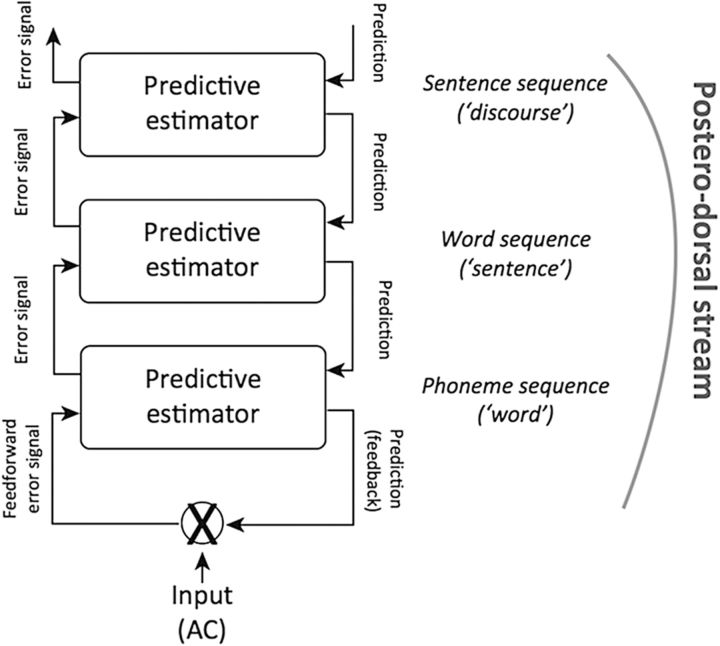
Predictions are propagated through the different levels from sentence through word to phoneme sequence processing. At the same time, prediction errors are propagated through the hierarchy in the opposite direction (Bornkessel-Schlesewsky et al., 2015). Reprinted with permission from Bornkessel-Schlesewsky et al. (2015).

A recent key insight is that, in neurobiological terms, language is processed in hierarchically organized temporal receptive windows (TRWs), whose function may be akin to receptive fields in the visual system (Lerner et al., 2011; for a parallel result in the visual system using movie stimuli, see Hasson et al., 2008). TRWs allow for processing at different timescales (e.g., sounds, words, sentences, and discourses), which increase from sensory to higher-order brain areas according to the principle of hierarchical organization (Hasson et al., 2015). A recent neurobiological model of higher-order language processing (Bornkessel-Schlesewsky and Schlesewsky, 2013; Bornkessel-Schlesewsky et al., 2015) posits that TRWs in the dorsal auditory stream provide the basis for a hierarchically organized predictive coding architecture (Friston et al., 2005; Friston and Kiebel, 2009), which generates predictions for upcoming input at different linguistic levels via a nested set of internal models (compare the proposal by Rao and Ballard, 1999 for the visual system). At longer timescales, these predictions are more abstract (e.g., for a particular part of speech at a certain position in a sentence), but at the shortest timescales they allow for matching to auditory input (i.e., the sequence of sounds identified as phonemes, combined into syllables and comprising the next word in the input). Prediction errors resulting from a mismatch between the predicted and actual sensory input are propagated up the model hierarchy, thereby leading to model adaptations (see Fig. 1). Thus, the dorsal stream, which connects auditory cortex to the posterior and dorsal part of the inferior frontal cortex via posterior superior temporal cortex, the inferior parietal lobule, and premotor cortex, provides the neurobiological infrastructure for predictive sequence processing at successively increasing timescales.

Evidence for linguistic sequence processing in the dorsal auditory stream has previously been observed from the level of sounds up to sentences (for review, see Bornkessel-Schlesewsky and Schlesewsky, 2013). However, a further crucial hypothesis following from the above account is that the dorsal auditory stream should show measurable effects of prediction at the discourse level, a hypothesized high-order window within the hierarchical organization of the dorsal auditory stream. While discourse processing has been investigated in previous fMRI studies, these have examined narrative processing (Xu et al., 2005), coherent and incoherent story comprehension (Kuperberg et al., 2006), narratives in the presence or absence of a title setting the discourse (Martín-Loeches et al., 2008) and the effect of discourse-setting words (e.g., Smirnov et al., 2014). By contrast, the hypothesis that the dorsal stream should show effects of predictive sequence processing at the discourse level remains untested to date. The aim of the present fMRI study was to test this hypothesis.

To test for discourse-based predictions, we used a story listening paradigm to examine the tracking of referents (persons, objects, etc.) within contextually rich narratives. Specifically, we examined the processing of identical human referents (e.g., a teacher or a journalist) depending on the story context in which they occurred and, more specifically, whether their previous mention in the story increased or decreased the predictability of a remention at a later point in time in the story. According to predictive coding, neural responses to predicted sensory input are attenuated (Rao and Ballard, 1999). Thus, if the hypothesis is correct, that the dorsal auditory stream implements a hierarchically organized predictive coding architecture for linguistic sequence processing, we should observe differences in BOLD amplitudes in this stream depending on how a referent was previously introduced into the discourse.

## Materials and Methods

### 

#### 

##### Participants.

Twenty-two monolingual native speakers of German participated in the study, all right-handed (Edinburgh Inventory of Handedness) (age mean = 24.3 years, SD = 2.1 years, male *N* = 6), recruited from postings at the University of Marburg. We excluded data from two participants due to movement artifacts, resulting in a total of 20 datasets. The study was approved by the ethics committee of the Faculty of Medicine of the University of Marburg. All participants gave written informed consent before participating in the study and were paid 10 Euros per hour for their participation.

##### Experimental design and stimuli.

To examine the neural correlates of predictive processing of a referent across a discourse, we created 20 stories in German, each of ∼2 min in length (±10 s; mean and SD of story length 306 (13) words, 23 (4) sentences). Within each story, we embedded the following sentence triplets: Sentence A introduced a subject referent in a manipulated context (described below), which was subsequently mentioned again in Sentence C. To tease apart the neural response to the referent in Sentence C from the response to its mention in Sentence A, an intervening Sentence B of variable length (mean: 4.30 s; SD: 0.96 s) acted as a natural jitter. Consider the following example (translated from the German original):

(1) “_…_ [The engineer pushed the pharmacist quickly back into the car,]_A_ [because due to the traffic one could not stay for long in the narrow street.]_B_ [*The engineer* sped off immediately.]_C …_”

In (1), Sentences A–C are indicated by square brackets and subscripts, with “the engineer” in Sentence C constituting the first mention of that referent after Sentence A. For convenience, we will refer to Sentence A as the “context sentence” in the following and the first subsequent mention of the subject (i.e., “the engineer” in Sentence C) as the “referent”. The measured responses concern the critical event of the referent.

Crucially, the way in which “the engineer” is introduced into the discourse in the context sentence can be manipulated to modulate the referent's predictability in the upcoming discourse. To draw attention to the sentence participant, which will be referred to in the upcoming discourse, the grammatical construction of passive voice is preferred in many languages (Primus, 1999; Gildea, 2012; Bornkessel-Schlesewsky and Schlesewsky, 2014). Text analyses across different languages (Givón, 1983, 1994) have demonstrated that subjects, especially the subjects of a passive sentence, have a higher tendency to reoccur in the following discourse as opposed to other grammatical roles (e.g., in “Peter hit Bill” and “Peter was hit by Bill,” “Peter” as the grammatical subject has a higher likelihood of mention in the following discourse than “Bill” and this likelihood is increased even further via the use of a passive construction, which involves an atypical subject choice). Therefore, we manipulated the context sentence to include a passive construction as in (2), after which the referent is more predictable because it has been introduced with a clear structural cue. The action of the sentence needed to be changed to keep the same entity in the subject position of an active versus passive voice: in (1) the engineer pushed the pharmacist, whereas in (2) the pharmacist pushed the engineer. We chose this design to keep the referent the same; in this way we could compare BOLD responses to the same referent, which had been introduced in a different discourse context.

(2) “_…_ [then the engineer was pushed quickly into the car by the pharmacist,]_A_ [because due to the traffic one could not stay for long in the narrow street.]_B_ [*The engineer* sped off immediately.]_C …”_

Importantly, referent tracking within a discourse is a multidimensional process. Thus, while some discourse-related factors, such as passivization, affect a referent's likelihood of subsequent mention (i.e., which of several possible referents the speaker will choose to refer to), others affect the choice of the referring expression used (e.g., whether the speaker will remention a previously introduced referent using a full noun phrase, such as *the engineer*, or a pronoun, such as *he*/*she*) (e.g., Kehler et al., 2008; Chiriacescu and von Heusinger, 2010; Kaiser, 2010). Use of a pronoun is typically viewed as indexing the high salience or accessibility of a referent, often conceptualized via the cognitive metaphor of “high activation.” Intuitively, this reflects the fact that a pronoun is used when the identity of a referent is salient enough to not warrant repetition (i.e., “he” or “she” suffices to for the hearer to realize that the speaker is referring to “the engineer”). Recent Bayesian approaches to referent tracking in discourse (e.g., Kehler et al., 2008; Kaiser, 2010) thus emphasize the need to distinguish the likelihood of referent remention, *P(referent*), the predictive component examined here, from the likelihood that, should a referent be rementioned, it will be expressed as a pronoun, *P(pronoun*|*referent*), a factor expressing the salience of a referent rather than its predictability. Referent salience, as indicated by *P(pronoun*|*referent),* is influenced by multiple grammatical and semantic factors (e.g., Arnold, 2001).

In our study, we always used full noun phrases rather than pronouns to refer back to the salient/nonsalient entity. Thus, while pronominalization is an important correlate of referent salience, it was not manipulated here. There were several reasons for this. First, the main focus of our study was on the neural correlates of predicting referent remention. Referent salience was included as a contrasting factor rather than one to be focused on in detail in its own right. Second, the comparison between pronouns and full noun phrases would have been rendered extremely difficult by the differences in length between the two (∼100 vs 600 ms, respectively). Thus, rather than manipulating the form with which a referent of a particular salience is rementioned, we manipulated the salience of the referent itself, and hence the likelihood of a full noun phrase being used in the event of a remention. In the spirit also adopted for our active/passive voice manipulation, we thereby kept the critical position constant while only manipulating the context leading up to it.

We manipulated referent salience via the causality of the event within which the critical referent was introduced. Highly causal events (e.g., *push*) tend to involve a highly prototypical event instigator (Agent) as well as a high prototypicality of the participant affected by the event (Patient). For low-causality events (e.g., *hold in high esteem*), the prototypicality of event participants is reduced. According to Dowty (1991), prototypical Agent properties are as follows: 1. volitional involvement in the event or state; 2. sentence (and/or perception); 3. causing an event or change of state in another participant; 4. movement (relative to the position of another participant); and 5. exists independently of the event named by the verb. By contrast, prototypical Patient properties are as follows: 1. undergoes change of state; 2. incremental theme; 3. causally affected by another participant; 4. stationary relative to movement of another participant; 5. does not exist independently of the event, or not at all. Low causality versions of examples (1) and (2) are given in examples (3) and (4).

(3) “_…_ [The pharmacist held the engineer in very high esteem.]_A_ [They knew each other for a long time so they had developed a strong/intimate friendship.]_B_ [*The pharmacist* was waiting in the car while the engineer was getting the tools from his apartment.]_C …_”

(4) “_…_ [The pharmacist was held in very high esteem by the engineer.]_A_ [They knew each other for a long time so they had developed a strong/intimate friendship.]_B_ [*The pharmacist* was waiting in the car while the engineer was getting the tools from his apartment.]_C …_”

High prototypicality of Agent and Patient participants increases referent salience, as indexed, for example, by pronominalization (e.g., Brown, 1983; for a recent replication in German, see Stein, 2016; Stevenson et al., 1994; for related findings, see Arnold, 2001). Additional evidence stems from visual word eye-tracking: Pyykkönen et al. (2010) used this technique to demonstrate that, even for young (3-year-old) children, both Agent and Patient are more salient after a highly causal action (e.g., a *pushing* event as in 1 and 2) than in a low causal situation (e.g., *being held in high esteem* as in examples 3 and 4 from the present study). By examining the total number of looks to Agent and Patient pictures, the authors found that children focused more frequently on the visual referents of high causality compared with low causality sentences. This result provides converging support for the link between verb causality and referent salience.

Together, these two manipulations resulted in a 2 × 2 design, including the factors voice (active vs passive) and causality (high vs low) with the following 4 conditions: AH: active voice and high causality (as in *hit*), AL: active voice and low causality (as in *hold in high esteem*), PH: passive voice and high causality (as in *was hit*), PL: passive voice and low causality (as in *was held in high esteem*). A schematic representation of the design is shown in Table 1. For our hypotheses, see the following subsection.

**Table 1.** The conditions refer to the discourse in which the referent was introduced*^a^*

	High causality	Low causality
Active voice	Active-high (AH) *pushed*	Active-low (AL) *held in high esteem*
Passive voice	Passive-high (PH) *was pushed*	Passive-low (PL) *was held in high esteem*

*^a^* BOLD response was measured on the remention of the referent in Sentence C.

Each of the 20 stories created included one instance of each of the four conditions; thus, four critical events on the referent (Sentence C) after the manipulated context sentence (Sentence A). The order of the conditions within a story was controlled such that all possible orders were realized. Furthermore, we controlled for the distance between the conditions to ensure that they did not always occur at the same time points across stories. For each story, there was a twin story in which the same verbs occurred but in the opposite voice, thus yielding 40 story stimuli in total.

The 40 stories were assigned to two experimental lists of 20 stories each such that only one version of each story was included in a single list. Each participant heard one list (randomized for each participant); thus, each participant heard one of the two versions of each story. The stories also contained additional manipulations, such as passages requiring Theory of Mind (ToM) processing. The ToM results from these datasets were previously published in Kandylaki et al. (2015).

Stimuli were spoken by a professionally trained female speaker of German at a normal speech rate. We recorded the stimuli in a sound proof EEG laboratory cabin with a sampling rate of 44.1 kHz and a 16 bit (mono) sample size. For sampling, we used the sound recording and analysis software Amadeus Pro (version 1.5.3, HairerSoft) and an Electret microphone (Beyerdynamic MC930C). The audio files of all stories are available in an online repository (Kandylaki, 2016b). A description of how the stories were constructed can be found in Kandylaki (2016a).

##### Hypotheses.

For the active/passive voice manipulation, assumed to modulate the predictability of a referent's remention, we expected to observe suppressed BOLD response levels for highly predicted referents (introduced via a passive sentence) versus less strongly predicted referents (introduced via an active sentence). This is in line with the well-known attenuation of neural activity in response to predicted sensory signals, as observed in multiple sensory domains (e.g., vision: Sylvester et al., 2008; audition: Curio et al., 2000; and somatosensation: Blakemore et al., 1998) and modeled in predictive coding architectures (e.g., Rao and Ballard, 1999; Friston, 2010). We further hypothesized that BOLD response differences at the position of the referent would manifest themselves most prominently in the dorsal auditory stream, effectively reflecting the processing of discourse-level referent sequences, as posited by the view that the dorsal stream processes temporal sequences in a hierarchically organized manner corresponding to temporal integration windows of successively increasing length (Bornkessel-Schlesewsky et al., 2015; Hasson et al., 2015). More specifically, this hypothesis is based on the assumption that passive-voice-induced predictions are not restricted to positing that the referent will reoccur at some unspecified time point in the future discourse. Rather, they can be considerably more precise about *when* the predicted remention of the referent might occur. Rementioned referents are discourse “topics,” which according to a robust linguistic generalization, tend to precede nontopical information and often occur sentence-initially (e.g., Clark and Haviland, 1977; for cross-linguistic confirmation, see Siewierska, 1993; for discussion from a sentence processing perspective, see e.g., Bock, 1982; Gernsbacher and Hargreaves, 1988). Thus, the predictability of a referent with a high likelihood of remention should increase at every new sentence beginning, cued in natural, auditory linguistic stimuli (as used in the current study) via the prosodic cues that signal a sentence boundary. As this amounts to a discourse-based prediction about *when in the current sequence of linguistic input* the referent is assumed to reoccur, we assume that these types of predictions should correlate with activation changes in the dorsal stream on account of that stream's importance for sequence processing at differing timescales (Bornkessel-Schlesewsky et al., 2015).

For the causality manipulation, assumed to modulate referent salience/accessibility, there are several possible competing hypotheses, depending on the assumed neurobiological implementation of referent salience. One possibility is that salience affects referent processing in a similar manner to effects of attention on stimulus processing, namely, by increasing rather than attenuating sensory signals (for a recent review, see Kok et al., 2012). In Friston and colleagues' active inference-based approach, attention is conceptualized as the mechanism that optimizes precision of sensory signals, which modulates the synaptic gain for prediction errors (Friston, 2009; Feldman and Friston, 2010). As demonstrated empirically by Kok et al. (2012), this can lead to a reversal of sensory attenuation for high precision stimuli (i.e., higher BOLD amplitudes for predicted vs unpredicted stimuli). From this perspective, we should expect to observe an interaction between voice and causality, with BOLD activity attenuated for referents in passive versus active low causality conditions, while this pattern should be reversed for the high causality conditions.

Alternatively, it may be the case that referent salience should not be conceptualized as akin to an attention-based modulation in neurobiological terms. Rather, it may reflect a complementary type of predictive process to that associated with the voice manipulation. Recall from the experimental design section that, rather than affecting the likelihood of a referent's remention, salience affects the form in which the referent will be expressed if it is mentioned again (e.g., as a pronoun; compare Kehler et al., 2008; Chiriacescu and von Heusinger, 2010; Kaiser, 2010). Consequently, it affects predictions of “what” (i.e., which stimulus type is expected), whereas voice affects predictions of “when” the referent might reoccur, as motivated above (compare Arnal and Giraud, 2012; Friston and Buzsáki, 2016), “topics” typically occur at the beginning of a sentence. From the perspective that our voice and causality manipulations both lead to predictive processing (albeit differing along the dimensions of “when” vs “what”), both should give rise to the sensory attenuation expected for predicted stimuli. As described above, however, no pronominal continuations were used in the present study to ensure that the critical referent was identical across all conditions. Thus, because all rementions were in the form of full noun phrases, the preference for a pronominal remention of a highly salient referent is never confirmed in our study. Therefore, a “what”-based prediction approach would lead us to expect higher BOLD responses (reflecting higher prediction errors) in the high versus low causality conditions for noun phrase remention of the referent.

Finally, when-based and what-based predictions should manifest themselves in separable networks. When-based predictions (voice) are expected to engender attenuated BOLD signals in regions associated with the processing of implicit timing (e.g., inferior parietal and premotor cortices) (for review, see Coull et al., 2011), these regions overlap with the dorsal auditory stream, thus rendering this hypothesis compatible with the first hypothesis formulated for the voice manipulation above. For what-based predictions (causality) by contrast, regions of expected activation change are somewhat more difficult to pinpoint given that the predictions in question involve a considerably higher level of abstraction than basic acoustic expectations. We might thus expect to observe activation in the ventral auditory stream in view of its importance in processing linguistic “auditory objects” (Rauschecker and Scott, 2009; DeWitt and Rauschecker, 2012; Bornkessel-Schlesewsky et al., 2015). Based on work in nonhuman primate audition (e.g., Rauschecker, 1998; Rauschecker and Tian, 2000), this perspective assumes that the ventral stream categorizes the speech input into perceptual and, at higher levels of abstraction, conceptual units: linguistic auditory objects. Thus, the ventral stream maps complex spectrotemporal patterns in the auditory input to meanings (Hickok and Poeppel, 2007; Saur et al., 2008; Rauschecker and Scott, 2009) rather than displaying the sensitivity to linguistic sequences manifested by the dorsal stream (Bornkessel-Schlesewsky and Schlesewsky, 2013; Bornkessel-Schlesewsky et al., 2015). Accordingly, auditory objects may provide an adequate approximation of the content of a “what”-based prediction.

##### Pretest.

We tested the stories before the imaging study to ensure their naturalness and the efficacy of the experimental manipulation. The pretests had two aims: (1) to test for the general properties of the stories (naturalness, probability, plausibility, and comprehensibility); and (2) to ensure that highly causal and low causal verbs indeed differed as intended in the manipulation.

In an internet-based questionnaire ratings from 177 participants were obtained (mean: 23.69 years, SD: 4.38 years, range: 18–58 years). We advertised the questionnaire through a students' mailing list. We discarded data of participants who did not fulfill the language criteria (monolingually raised German native speakers). Because of the auditory presentation of the stories, we strongly encouraged the participants to use earphones for completing the questionnaire. To keep the amount of time needed for questionnaire completion at ∼20 min, each participant was asked to rate two stories, which were presented in four or five consecutive parts (depending on the story). Naturalness, probability, plausibility, and comprehensibility of the stimuli were assessed through the following questions (translated from the German original): “How natural was this passage?”; “How probable is the event that was described in the passage?”; “How often does this happen?”; “How comprehensible was this passage?”. When applicable (i.e., when the current part of the story included a causal manipulation), they also rated the agent and undergoer features (Dowty, 1991) with the Kako (2006) questions translated into German. These properties have been tested before using offline (Kako, 2006) and online paradigms (Pyykkönen et al., 2010). Ratings were collected on a 4-point scale, on which 1 was the lowest value and 4 the highest value. Table 2 shows the mean and SD of all ratings for all conditions.

**Table 2.** Mean (SD) of ratings for naturalness, probability, plausibility, and comprehensibility as well as for event causality quantified in the Dowty (1991) proto-role properties*^a^*

Condition	NAT	PRO	PLA	COM	VOL	SEN	CAU	MOA	MOU	CHA	CHU
AH	3.2 (0.8)	3.0 (0.9)	2.6 (0.8)	3.6 (0.6)	3.6 (0.8)	3.6 (0.7)	3.3 (0.9)	3.6 (0.8)	1.8 (0.9)	3.2 (0.9)	3.3 (0.8)
AL	3.2 (0.8)	2.9 (0.9)	2.5 (0.8)	3.6 (0.7)	3.0 (1)	3.0 (0.9)	2.7 (1)	2.8 (1)	2.2 (1)	2.7 (1)	2.7 (1)
PH	3.2 (0.8)	2.9 (0.9)	2.5 (0.8)	3.6 (0.6)	3.5 (0.9)	3.6 (0.7)	3.2 (0.9)	3.6 (0.8)	1.8 (0.9)	3.3 (0.8)	3.3 (0.8)
PL	3.1 (0.8)	2.9 (0.9)	2.5 (0.8)	3.6 (0.6)	3.2 (1)	3.1 (0.9)	2.9 (1)	2.9 (1)	2.2 (1)	2.9 (1)	2.9 (1)

*^a^* NAT, Naturalness; PRO, probability; PLA, plausibility; COM, comprehensibility; VOL, volition; SEN, sentience; CAU, causation; MOA, motion agent; MOU, motion undergoer; CHA, change agent; CHU, change undergoer; AH, active-high; AL, active-low; PH, passive-high; PL, passive-low.

The results of the questionnaire pretest were analyzed using linear mixed effects models (package lme4, Bates et al., 2014) in R (Team, 2014). To assess the effects of the different factors on the ratings, we used a forward model selection procedure within which we used likelihood ratio tests to compare a base model, including only an intercept with successively more complex models, including voice and causality as fixed factors. Participants and stories were modeled as random factors (Barr et al., 2013). Only random intercepts by participant and story were included due to convergence problems with more complex random effects structures. For simplicity, in what follows, we will refer to the model with only main effect of causality plus random factors as “causality model,” to the model with only main effect of voice plus random factors as “voice model,” to the model with only random factors as “null model,” to the model with main effects of both causality and voice as “main effects model” and to the model of the interaction of causality and voice as “interaction model.” All reported *p* values are outputs of the ANOVA function in R, which we used for pairwise model comparisons.

The mixed-model analyses showed no significant differences between conditions for naturalness, probability, plausibility, and comprehensibility. The null model was the minimal adequate model for the tested general properties of the stimuli (for the *p* values of the model comparisons, see Table 3).

**Table 3.** *p* values of the model comparisons for the questionnaire ratings*^a^*

Comparison	NAT	PRO	PLA	COM	VOL	SEN	CAU	MOA	MOU	CHA	CHU
N versus C	0.198	0.669	0.865	0.171	<0.001	<0.001	<0.001	<0.001	<0.001	<0.001	<0.001
N versus V	0.521	0.238	0.722	0.917	0.770	0.931	0.662	0.096	0.294	0.025	0.020

*^a^* N versus C is the comparison of the null model (only random factors) to the model with main effect of causality. N versus V is the comparison of the null model (only random factors) to the model with main effect of voice. NAT, Naturalness; PRO, probability; PLA, plausibility; COM, comprehensibility; VOL, volition; SEN, sentience; CAU, causation; MOA, motion agent; MOU, motion undergoer; CHA, change agent; CHU, change undergoer.

For the causality ratings, we found that the causality model provided the best fit for the agent features volition, sentience, causation, as well as for motion of undergoer (for the inferential statistics, see Table 3). For motion of agent, there was a marginal improvement of fit for the voice model compared with the null model (Table 3), but between the causality and the main effects model, there was no significant difference (*p* = 0.127). For change agent and change undergoer, we found that the model, including main effects of both causality and voice, showed a significantly improved fit over the simpler models (for comparison of main effect of causality and main effect of voice with null model, see Table 3) and the more complex models (change agent: comparing causality model and main effects model *p* = 0.033, comparing voice model and main effects model *p* < 0.001, comparing main effects to interaction model *p* = 0.939; change undergoer: comparing causality model to main effects model *p* = 0.027, comparing voice model to main effects model *p* < 0.001, comparing main effects to interaction model *p* = 0.755).

##### Acoustic analysis.

To determine whether our critical conditions differed in prosodic terms, we performed an acoustic analysis of the context sentence (Sentence A) and the referent (beginning of Sentence C). We extracted measurements for duration, sound intensity, and average frequency (pitch), as these are the parameters used to modulate intonation in speech production. Table 4 presents the descriptive statistics of all measures. After checking the distribution of the measures using the allfitdistr function in MATLAB (The MathWorks), we discovered that we could not fit general or generalized linear models to our data because the data followed different distributions that cannot be modeled with current glmer options in R. Therefore, we performed the inferential statistics using the Kruskal–Wallis test in which the dependent variable is numeric (matched our measures) and the independent variable is a factor. Table 5 shows the results of the Kruskal–Wallis tests.

**Table 4.** Mean (SD) of duration, average intensity, and average frequency for the context sentence and for the referent*^a^*

Condition	Context sentence				Referent			
	AH	AL	PH	PL	AH	AL	PH	PL
Duration (in seconds)	2.854 (0.820)	2.559 (0.710)	3.232 (0.864)	3.053 (0.737)	0.610 (0.131)	0.615 (0.163)	0.589 (0.144)	0.609 (0.163)
Average intensity (in dB)	48 (2)	48 (2)	48 (2)	48 (2)	51 (4)	51 (4)	51 (4)	52 (4)
Average frequency (in Hz)	185 (9)	185 (8)	185 (9)	183 (9)	197 (16)	197 (18)	195 (17)	205 (18)

*^a^* AH, Active-high; AL, active-low; PH, passive-high; PL, passive-low.

**Table 5.** Inferential statistics for the acoustic analysis (*p* values of Kruskal–Wallis tests)

Criterion	Context sentence		Referent	
	By voice	By causality	By voice	By causality
Duration	0.0006236	0.06314	0.6818	0.515
Average intensity	0.5898	0.8618	0.4779	0.3505
Average frequency	0.5572	0.6872	0.3262	0.07777

For the context sentence, the acoustic parameters of our critical conditions did not differ significantly with regard to average intensity and average frequency. They differed in duration, showing effects of both voice and causality. The voice effect is expected given that the passive sentences were ∼500 ms longer than the active sentences. syntactical structure crossed with the causality of the described event. Importantly, however, we used the intervening sentence between context sentence and referent (Sentence B) to introduce a natural jitter, thus allowing us to separate the brain response to the referent from that to the context sentence. Therefore, it appears unlikely that any activation differences related to the acoustic parameters of the context sentence would have been carried over to the processing of the referent.

For the referent, duration and average intensity did not reveal any significant effects of voice or causality. For average frequency, voice did not have a significant effect, whereas causality showed a marginal significance for frequency differences. This marginal effect of causality on frequency involved differences in the order of 2.5% of the average frequency. It is arguable whether such small differences are perceivable in speech. According to Rietveld and Gussenhoven (1985), differences of <3 semitones are generally not perceivable in speech comprehension. The average frequency of high causality referents differed less than a semitone from that of low causality referents; therefore, the marginal effects in the acoustic analysis cannot have influenced the behavioral results. However, from this, it does not necessarily follow that the brain is insensitive to such differences. Also, other acoustical features (such as the F_0_ dynamics or the F_0_ variance, which have not been analyzed here) might have driven a part of variance of the BOLD response, thereby possibly confounding the prosodic aspects with the conditions of interest. However, in contrast to the highly controlled materials used in traditional studies, the contextually rich, naturalistic auditory stimuli used in the present study render it inherently difficult, if not impossible, to fully control for prosodic differences between conditions.

##### Imaging procedure and behavioral data acquisition.

Participants completed a training session before entering the scanner for the fMRI session. In the training session, they listened to two stories (not part of the experimental stimuli) and answered to two questions after each story. The fMRI session included 20 stories and 40 questions (two after each story). The stories were presented auditorily through MRI-compatible earphones, and the questions were presented visually, along with their possible answers. For each participant, we optimized loudness before the start of the experiment. The presentation order of the stories was randomly assigned for each participant, to avoid sequence effects. The stories were presented in 4 blocks of 5 stories each. Between two blocks, there was a break of 45 s. During the break, the visual message “Short break!” was shown in the middle of the screen while the scanner was still running.

Each story trial was structured as follows: Before the start of each story, a fixation cross was shown in the middle of the screen for 500 ms. The cross was then replaced by a fixation point to indicate the beginning of the story which lasted ∼2 min. After the story, we included a jitter between 1.5 and 3 s (duration assigned randomly) followed by the visual presentation of the first question (referring to the immediately previous story). The question asked either about a specific person or object mentioned in the story, which were involved in a different situation than the ones described by the current critical manipulations (e.g., “How many sandwiches were left for the woman, after the man had finished eating?”). The whole question was presented at once, centered and toward the top of the screen for 5 s before the possible answers appeared toward the bottom of the screen for a maximum duration of 3 s, clearly separated from each other; each answer was marked with an index letter a (always on the left) and b (always on the right side of the screen below the question) (example answers for the previous question: “One” vs “Three”). After the first question, a second question followed with the same presentation procedure and similar type of content as the first question. Participants gave their answers by pressing the left or right button on a customized button box, fixed to their left leg, with their left middle or index finger accordingly. The left hand was chosen as a response hand to minimize left hemispheric artifacts, which could overlap with language processing (Callan et al., 2004). The position of the correct answer was counterbalanced across the experiment. All visual stimuli (cross, fixation point, questions and answers) were presented in dark gray foreground on light gray background.

Presentation of stimuli was time-jittered between story and questions and also between first and second question. The whole procedure was implemented and presented using the software package Presentation (Neurobehavioral Systems).

##### fMRI data acquisition.

During the MR session, a series of echo-planar-images recorded the time course of the subjects' brain activity. Measurements were performed on a 3 tesla MRI system (Trio, A Tim System 3T, Siemens) with a 12-channel head matrix receive coil. Functional images were acquired using a T2*-weighted, single-shot EPI sequence: parallel imaging factor of 2 (GRAPPA), TE = 25 ms, TR = 1450 ms, flip angle 90°, slice thickness 4.0 mm and 0.6 mm gap, matrix 64 × 64, field of view = 224 × 224 mm, in-plane resolution 3.5 × 3.5 mm^2^, bandwidth 2232 Hz/pixel, EPI factor of 64, and an echo spacing of 0.53 ms. Transversal slices oriented to the AC-PC line were gathered in ascending order.

The initial five images were removed from the analyses to avoid saturation and stabilization effects. Head movements of the participants were minimized by using foam paddings.

A whole-head T1-weighted dataset was acquired with a 3d MPRAGE sequence: parallel imaging factor of 2 (GRAPPA), TE = 2.26 ms, TR = 1900 ms, flip angle 9°, 1 mm isometric resolution, 176 sagittal slices, 256 × 256 matrix.

##### fMRI data analysis.

All analyses for the fMRI data were calculated in SPM8 (Wellcome Trust Centre for Neuroimaging), implemented in MATLAB.

A slice time correction (to the 15th slice) was performed first. Then images were realigned to the first image to correct for head movement artifacts. We then normalized the volumes into standard stereotaxic anatomical MNI space by using the transformation matrix calculated from the first EPI scan of each subject and the EPI template. On the normalized data (resliced voxel size 2 mm^3^), we applied an 8 mm FWHM Gaussian smoothing kernel to compensate for intersubject anatomical variation.

For the single-subject analysis, we created the design matrix for each individual subject based on the presentation log files because each participant heard the stories in a different order. We modeled the critical events in seconds: mean duration 0.604 s and SD 0.152 s. As factors of no interest, we modeled separately: the rest of the stories excluding Sentence A and the referent on Sentence C (speech), Sentence A, the question, the answer, the button presses and the jitters before each question. The middle sentence between manipulated context and referent (Sentence B) had a mean duration of 4.3 s and a SD of 0.96 s: this was not modeled separately, but it was included in the regressor for speech (which excluded Sentence A and referents). Our baseline consisted of the 45 s pauses between blocks. To remove movement artifacts for each individual session, the realignment parameters were entered as separate regressors in the first-level analysis.

In the group-level analysis, we modeled the BOLD response to the referent in the 2 × 2 factorial design of Table 1 for the first-level conditions: AH versus baseline, AL versus baseline, PH versus baseline, and PL versus baseline. Brain activations were plotted on the anatomical MRIcroN template. We used the cluster extent thresholding algorithm by Slotnick et al. (2003), which implements an FWE correction using a Monte Carlo simulation approach, to correct for multiple comparisons. We set the desired FWE correction for multiple comparisons to *p* < 0.05 and the assumed voxel type I error to *p* < 0.005; after 10,000 iterations, our cluster threshold was 72 voxels. For all fMRI results reported here, we thus used an individual voxel threshold of *p* = 0.005 and cluster extend threshold of 72 voxels in a whole-brain analysis.

For the localization of the effects, we followed a three-step procedure to ensure that the provided neuroanatomical details are as accurate as possible. First, we ran the SPM anatomy toolbox on the results matrices, which outputted the descriptions of the clusters as well as one label for the peak voxel of each cluster. Second, we used AFNI's command line tool “whereami,” which takes MNI coordinates as input and returns labels for the peaks based on current anatomy atlases (e.g., Talairach-Tournoux Atlas, Eickhoff-Zilles cytoarchitectonic maximum probability map on MNI-152 from postmortem analysis, the atlas used by the SPM anatomy toolbox) (Caspers et al., 2006; Eickhoff et al., 2007; Bludau et al., 2014) and the maximum probability maps of Destrieux et al. (2010). Third, we reported the label of the peak voxel as labeled by the SPM anatomy toolbox and a summary of the outputs of different atlases in the description of the anatomical region of each cluster.

## Results

Subjects achieved 90% (SD = 5.61) correctness in their answers to the comprehension questions after each story. This is an indicator of their attentive listening during the auditory story presentation.

The interaction between voice and causality showed suprathreshold activation in the left supplementary motor area (SMA), extending to medial parts of the superior frontal gyrus. The main effect of voice was localized in 13 clusters with local maxima in the right inferior parietal lobule (IPL), middle cingulate cortex bilaterally, left anterior cingulate cortex (ACC), middle and inferior frontal gyrus (MFG, IFG) bilaterally, cerebellar vermis (VER) and left cerebellum (CE) IV–V. The main effect of causality showed suprathreshold activation in 5 clusters which had their local maxima in the middle temporal gyrus (MTG) bilaterally, left middle occipital gyrus, and left fusiform gyrus. Table 6 shows the coordinates and statistics of the peak voxels for the referent analysis.

**Table 6.** Clusters with their local maxima coordinates and neuroanatomical details of their extend for the interaction (INT) of voice and causality and for the main effects (MEF) of voice and causality (*p* < 0.005, cluster threshold 72 voxels, Monte Carlo corrected)*^a^*

Contrast	Anatomical region	H	MNI coordinates	F	Cluster size in voxels
INT voice and causality	**Supplementary motor area (SMA):** cluster extending from BA 6 to the medial part of the focus point in left SMA	L	−8, 14, 66	18.45	86
MEF voice	**Inferior parietal lobule (IPL):** activation between SMG (PFm) and AG (PGa) with larger portion of cluster in the SMG (37.7%) as opposed to AG (19.5%); maximum lies within the SMG	R	50, −50, 44	17.10	405
	**Middle cingulate cortex (MCC):** medial activation within the middle part of the cingulate cortex, focus point left MCC	L	−6, 26, 36	16.13	158
	**Middle cingulate cortex (MCC):** MCC activation extending in the superior posterior direction into the right BA 4a	R	8, −18, 36	15.86	447
	**Middle frontal gyrus (MFG):** right MFG activation, extending from the superior part of BA 9 to the inferior BA 8	R	42, 28, 42	15.70	405
	**Middle frontal gyrus (MFG):** superior and middle frontal gyrus activation; the peak voxel falls within the MF, but the extent of the cluster lies mostly in the superior frontal gyrus, specifically right BA 8	R	30, 18, 56	15.65	210
	**Middle frontal gyrus (MFG):** cluster located in the right MFG and specifically frontal pole cytoarchitectonic area Fp1	R	26, 56, 24	14.42	276
	**White substance (close to midbrain):** basal ganglia cluster	R	8, 2, −10	14.28	73
	**Inferior frontal gyrus (IFG):** IFG activation (BA 45), with maxima in ORBinf and IFGtriang	R	32, 24, −12	14.07	351
	**Cerebellum (CE) IV–V:** left cerebellar activation extending from lobules V and VI to the left culmen	L	−20, −48, −20	13.47	128
	**Inferior frontal gyrus (IFG):** left IFG cluster extending to the MFG with maxima in frontal pole, pars triangularis, and middle orbital gyrus	L	−48, 42, 8	13.42	192
	**Anterior cingulate cortex (ACC):** left ACC cluster, activating the cingulate subregion s32 and extending to the left MFG	L	8, 42, −4	13.35	223
	**Cerebellar vermis (Ver):** cerebellar vermis activation, specifically in the nodule		−4, −58, −32	12.57	132
	**Middle frontal gyrus (MFG):** left MFG activation, located toward the superior part of the MFG and extending to BA 8 and 9	L	−36, 26, 38	12.06	99
MEF causality	**White substance close to insular lobe (INS):** cluster localized between white matter tracts and insular lobe extending to the right IFG	R	30, 12, −12	18.69	239
	**Middle temporal gyrus (MTG):** cluster extending between the right middle temporal, middle occipital, and angular gyri with maximum in the MTG	R	50, −66, 8	18.12	225
	**Middle occipital gyrus (MOG):** cluster extending between the left angular and middle occipital gyri with its maximum in the MOG	L	−34, −88, 30	17.24	81
	**Middle temporal gyrus (MTG):** cluster within the MTG and maximum in the MTG	L	−56, −38, −6	14.81	148
	**Fusiform gyrus (FFG):** cluster within the fusiform gyrus with parts of it extending to the ITG and maximum in the FFG	L	−42, −54, −14	12.84	112

*^a^* Coordinates are listed in MNI atlas space. H, Hemisphere; BA, Brodmann area; PFm, subregion of the supramarginal gyrus (SMG) (for cytoarchitectonic features, see Caspers et al., 2006); PGa, rostral subregion of the angular gyrus (AG) (for cytoarchitectonic features, see Caspers et al., 2006); Fp1, subregion of the frontal pole (for cytoarchitectonic features, see Bludau et al., 2014); ORBinf, orbital part of the inferior frontal gyrus (IFG); IFGtriang, triangular part of the IFG (as abbreviated by Tzourio-Mazoyer et al., 2002).

**Figure 2.**
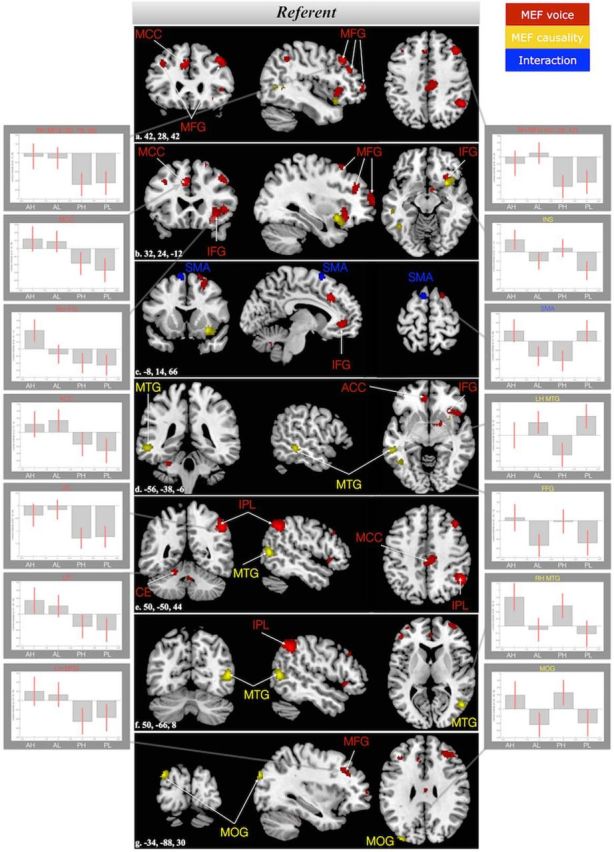
Supra-threshold clusters (*p* < 0.005, cluster extend threshold 72 voxels) at the referent. Red represents the clusters for the main effect of voice. Yellow represents the clusters for the main effect of causality. Blue represents the cluster of the interaction. The slice numbers are included in the left bottom corner of the brain maps a–g. ACC, anterior cingulate cortex; AH, active high; AL, active low; CE, cerebellum; FFG, fusiform gyrus; IFG, inferior frontal gyrus; INS, insula; IPL, inferior parietal lobe; LH, left hemisphere; MCC, middle cingulate cortex; MEF, Main effect of; MFG, middle frontal gyrus; MOG, middle occipital gyrus; MTG, middle temporal gyrus; PH, passive high; PL, passive low; RH, right hemisphere; SMA, supplementary motor area.

Figure 2 shows the localization of the results on the referent, as plotted on the anatomical high resolution template of MRIcroN (the Colin brain); also, the contrast estimates (plus 90% confidence intervals) for all four conditions are included in the subpanels. The direction of the main effect of voice in all activated regions follows our hypothesis: passive voice conditions (PH, PL) showed an attenuated BOLD response compared with active voice conditions (AH, AL). Notably, in addition to being reduced compared with the BOLD responses for the active conditions, the BOLD signals for the passive conditions were negative-going compared with the baseline in all regions showing a main effect of voice.

For the main effect of causality on the referent, the activated areas can be divided into two groups depending on their BOLD response patterns. Higher BOLD responses to the referent after high versus low causality were found in the left MOG, left FFG, and right INS. The opposite pattern was found for the MTG bilaterally: higher BOLD responses to referents after a low causality context sentence than after a high causality one.

**Figure 3.**
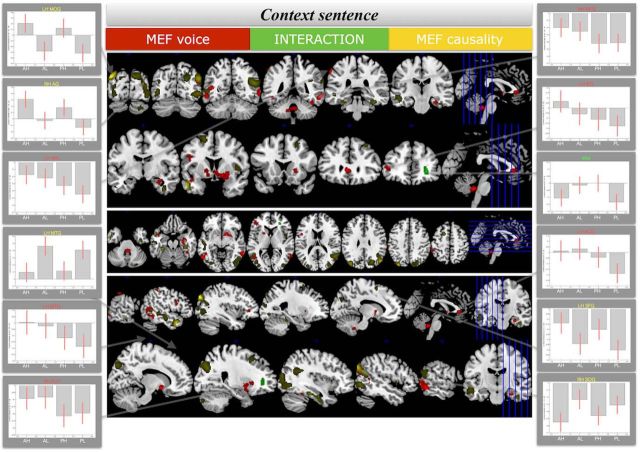
Supra-threshold clusters (*p* < 0.005, cluster extend threshold 72 voxels) at the context sentence (Sentence A). Red represents the clusters for the main effect of voice. Yellow (and its darker shades) represents the clusters for the main effect of causality. Green represents the cluster of the interaction. ACC, anterior cingulate cortex; AG, angular gyrus; AH, active high; AL, active low; IFG, inferior frontal gyrus; LH, left hemisphere; MEF, Main effect of; MOG, middle occipital gyrus; MTG, middle temporal gyrus; PH, passive high; PL, passive low; PUT, putamen; RH, right hemisphere; SFG, superior frontal gyrus; SOG, superior occipital gyrus; SPL, superior parietal lobule; WM, white matter.

Finally, to make available a comparison of the responses to the manipulated context sentence (Sentence A) and to the referent in Sentence C, Figure 3 provides an overview of the BOLD responses to the manipulated context sentence. Even though the direction of the effects is the same to that observed at the position of the Referent, BOLD response changes were observed in different regions (left IFG, left ACC, left putamen, bilateral MTG, and right temporal pole), distinct from those for which we observed activation changes at the position of the referent. In addition, the pattern of effects as evidenced by the bar graphs does not show the same clear, below-baseline attenuation of responses for passive conditions only, as was the case at the position of the referent. The only potential dorsal auditory region for the main effect of voice for the manipulated sentence, is the left SPL, but this region is again distinct from those observed for the voice manipulation at the position of the referent. More details on the clusters and on the localization of the peak voxels for the context sentence can be obtained from the corresponding author on request.

## Discussion

The present study investigated the neural correlates of discourse prediction and salience in auditory stories. Predictability of the referent was manipulated in the structure of the context sentence, presented two sentences before the occurrence of the referent. Based on prior linguistic research, we hypothesized that the referent would be more predictable after a passive voice construction and that this effect would manifest itself within the human dorsal auditory stream within a hierarchical predictive coding architecture (Friston et al., 2005; Friston and Kiebel, 2009). We also manipulated referent salience via the type of event described in the context sentence (e.g., hitting event vs holding in high esteem situation). Based on former psycholinguistic findings (Kako, 2006; Pyykkönen et al., 2010), referents are more salient after a highly causal event compared with a low causal event.

### Predicting referents: predictive coding of sequences (“when”) at the discourse level

For the voice manipulation, our results followed the expected pattern of activation along the dorsal stream (right IPL, bilateral MFG, left IFG; pars triangularis) and the expected direction: the passive conditions invariably showed a reduced BOLD response compared with the baseline in all regions manifesting a main effect of voice. This pattern is highly compatible with the sensory attenuation of a predicted stimulus as assumed in predictive coding approaches (i.e., silencing of prediction errors via inhibition of expected sensory inputs) (e.g., Rao and Ballard, 1999; Friston, 2005). Our results thus provide converging support for the assumption that the dorsal auditory stream plays an important role in predictive sequence processing at the discourse level (Hasson et al., 2015; compare Bornkessel-Schlesewsky et al., 2015).

Turning now to a more specific discussion of the regions that showed a main effect of voice, the observed effect in the IPL is not only viewed as part of the dorsal stream but is also consistent with previous studies on discourse processing (Xu et al., 2005, Martín-Loeches et al., 2008, Smirnov et al., 2014), which have consistently reported IPL (SMG/AG) activation in connection to global coherence processes (Martín-Loeches et al., 2008, Smirnov et al., 2014). The observation of SMG activation in the present study may be tied to the temporal prediction involved in the voice contrast (predicting “when”): activation of left SMG was identified as the common denominator of implicit timing tasks in a recent meta-analysis (Wiener et al., 2010). Implicit timing refers to scenarios in which timing is important, but is not focused explicitly by the task (Coull and Nobre, 2008), as is the case in the present study.

For the main effect of voice, we also observed activations in regions outside of the dorsal auditory stream. These include the cingulate gyrus (MCC, ACC) and the cerebellum (IV–V). It appears plausible to assume that the cerebellar activation is linked to temporal predictions (predicting “when”), as the cerebellum is known to play an important role in timing, including temporal expectations (Coull et al., 2011). ACC activation, by contrast, has been connected to cognitive control and decision-making (Shenhav et al., 2013). In connection to our manipulation of voice as a predictive cue, the ACC, insula, and cerebellum activation might be interpreted as subserving top-down, discourse-based modulations of sensorimotor predictions during speech perception (Hickok et al., 2011).

### Referent salience: attention or “what”-based prediction?

Salience of referents was manipulated by the type of event described in the context sentence (e.g., hitting event vs holding in high esteem situation). Rather than affecting the likelihood of referent remention (as for the voice manipulation), salience affects the likelihood of how the referent will be expressed if it is rementioned (e.g., as a pronoun; Kehler et al., 2008; Chiriacescu and von Heusinger, 2010; Kaiser, 2010), although we did not manipulate the form of the referent remention. We suggested two alternative hypotheses regarding how salience might manifest itself neurobiologically: (a) attention-based, thereby affecting the precision/synaptic gain of prediction errors and reversing their effects for attended versus unattended stimuli (Kok et al., 2012); (b) by modulating predictions of “what” as opposed to predictions of “when,” as effected by the voice manipulation. Whereas hypothesis (a) predicted an interaction between voice and causality, hypothesis (b) predicted main effects of voice, with higher BOLD responses for high versus low causality conditions.

Hypothesis (a) was not borne out in our data: although we did observe an interaction between voice and causality in the left SMA, this showed the opposite pattern to what would have been expected if referent salience had similar effects to attention in a predictive coding framework. Rather than observing an attenuated BOLD response for the highly versus less predicted condition under low attention/salience (passive low causality, PL < active low causality, AL) and the reversed pattern for high attention/salience (passive high causality, PH > active high causality, AH), the SMA showed the inverse response pattern (PL > AL and PH < AH).

Hypothesis (b), by contrast, was partially borne out in that several regions showed the expected activation pattern of higher BOLD responses for high versus low causality conditions: left MOG, left FFG, and right INS. While the MOG and FFG in particular do not fall into the ventral auditory stream, in which we expected to observe activation differences under this hypothesis, these regions are nevertheless involved in semantic processing of speech (for review, see Price, 2012). From a *post hoc* perspective, they are thus consistent with the assumption of predicting “what” (i.e., type of referential form used) in regard to referent tracking. The observation of activation differences in left ventral occipitotemporal regions in this context is also in line with previous observations of increased activation in these regions, and specifically the left FFG, for repeated names in discourse, as opposed to names referred to by a pronoun upon remention (Almor et al., 2007). Nevertheless, future research will need to explore why this contrast engendered activation in regions that form part of the ventral visual rather than auditory stream, despite the use of auditory story stimuli.

In contrast to the expected directionality of BOLD responses in the left MOG, left FFG, and right INS, bilateral posterior MTG showed the opposite pattern: higher BOLD responses to referents introduced in low causal events as opposed to highly causal events. Posterior MTG has often been associated with “lexical” processing (i.e., retrieval of words and associated information from memory) (see, e.g., Hickok and Poeppel, 2007; Hagoort, 2014). More salient referents (introduced in a highly causal event) may be easier to retrieve and thus expected to show lower BOLD responses compared with nonsalient referents (introduced in a low causal event). We acknowledge, however, that this *post hoc* explanation is orthogonal to the predictive coding-based framework adopted throughout the remainder of this paper.

### Implications: predicting “when” versus “what” in discourse

From the above discussion, it is apparent that the most parsimonious account for the present results, and in particular for both the voice and causality manipulations, is prediction-based. Active versus passive voice affects the probability for remention of a referent, thereby modulating a “when”-based prediction. Event causality, by contrast, affects referent salience and thereby modulates the probability for which form a referent will be mentioned in if it does reoccur in the discourse, amounting to a prediction of “what” (content rather than timing). We observed that when- and what-based predictions (compare Arnal and Giraud, 2012; Friston and Buzsáki, 2016) engaged separable neural networks: an extended dorsal stream network also incorporating timing-relevant regions, such as the cerebellum and a ventral occipitotemporal network, respectively. They also showed dissociable BOLD response patterns in that when-based predictions were reflected in the expected pattern of BOLD attenuation for predicted stimuli, whereas the what-based predictions manifested as increased prediction errors (higher BOLD responses) for highly salient referents in the present study on account of the absence of pronominal forms for remention of the referent. Notably, the when-based prediction view is highly compatible with the perspective that the dorsal auditory stream engages in hierarchically organized predictive sequence processing in successively longer TRWs, here spanning between three sentences. The regions observed in response to the manipulation of what-based predictions, by contrast, was not straightforwardly derivable on the basis of the existing literature and will require further investigation in future research.

Together, these results suggest that the neural basis of language processing within rich contexts draws upon very similar mechanisms to those assumed for the processing of smaller linguistic units. Based on the current study, one possible “extension” to the so-called “extended language network,” which has been discussed as requiring the recruitment of additional regions for the purposes of text and discourse comprehension (e.g., Ferstl et al., 2008; Hagoort, 2014), would be the incorporation of how longer TRWs are processed, thus operating under a predictive coding mechanism along the dorsal auditory stream. Moreover, rather than being specific to language, such a mechanism is applicable to multiple cognitive domains, thus supporting the proposal that neurobiological models of language processing should build on neurobiologically plausible mechanisms of information processing in the brain (Bornkessel-Schlesewsky et al., 2015).
